# Icariin Metabolism by Human Intestinal Microflora

**DOI:** 10.3390/molecules21091158

**Published:** 2016-08-31

**Authors:** Hailong Wu, Mihyang Kim, Jaehong Han

**Affiliations:** Metalloenzyme Research Group and Department of Integrative Plant Science, Chung-Ang University, Anseong 17546, Korea; a550539053@hotmail.com (H.W.); mihcoterie@gmail.com (M.K.)

**Keywords:** biotransformation, desmethylicaritin, *Epimedium koreanum*, human intestinal bacteria, icariin, icariside II, icaritin

## Abstract

Icariin is a major bioactive compound of Epimedii Herba, a traditional oriental medicine exhibiting anti-cancer, anti-inflammatory and anti-osteoporosis activities. Recently, the estrogenic activities of icariin drew significant attention, but the published scientific data seemed not to be so consistent. To provide fundamental information for the study of the icaritin metabolism, the biotransformation of icariin by the human intestinal bacteria is reported for the first time. Together with human intestinal microflora, the three bacteria *Streptococcus* sp. MRG-ICA-B, *Enterococcus* sp. MRG-ICA-E, and *Blautia* sp. MRG-PMF-1 isolated from human intestine were reacted with icariin under anaerobic conditions. The metabolites including icariside II, icaritin, and desmethylicaritin, but not icariside I, were produced. The MRG-ICA-B and E strains hydrolyzed only the glucose moiety of icariin, and icariside II was the only metabolite. However, the MRG-PMF-1 strain metabolized icariin further to desmethylicaritin via icariside II and icaritin. From the results, along with the icariin metabolism by human microflora, it was evident that most icariin is quickly transformed to icariside II before absorption in the human intestine. We propose the pharmacokinetics of icariin should focus on metabolites such as icariside II, icaritin and desmethylicaritin to explain the discrepancy between the in vitro bioassay and pharmacological effects.

## 1. Introduction

**Figure 1:**
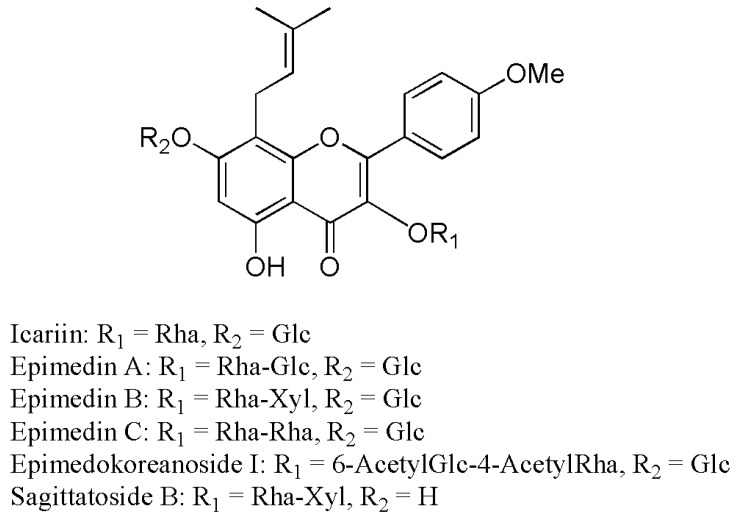
Molecular structures of the major prenylflavonoids isolated from Epimedii Herba.

Epimedii Herba is the aerial part of the plant species belonging to the genus *Epimedium*, including *E. koreanum* Nakai, *E. brevicornum* Maximowicz, *E. pubescens* Maximowicz, *E. wushanense* T. S. Ying, and *E. sagittatum* Maximowicz (Berberidaceae). It is also known as horny goat weed or yin yang huo and is listed as an herbal medicine in the pharmacopeias of Korea, Japan and China. It has been traditionally used for various biological activities, such as treating impotence, delaying aging, enhancing bone formation, and improving the function of the cardiovascular system [1]. A major bioactive ingredient of Epimedii Herba is prenylflavone glycosides with an 8-prenyl group on the kaempferol skeleton. Until now, more than 20 derivatives of 8-prenylkaempferol 3,7-*O*-diglycosides, including icariin, epimedin A, epimedin B, epimedin C, epimedokoreanoside I, and sagittetoside B, have been reported from *E. koreanum* (Figure 1) [2].

Icariin is the index compound of Epimedii Herba (KP), and the biological activities, pharmacological effects, pharmacokinetics and metabolism of icariin were extensively studied in the animal models [3]. Along with the immune-enhancing effects [4], the phytoestrogenic activity [5] and osteogenic effects [6,7] of icariin were also reported. However, the estrogenic activity of icariin did not directly correlate with the expected pharmacological effects, such as the anti-osteoporosis and neuroprotective activities measured at different levels of experiments [8,9]. For example, icariin was the weakest phytoestrogen compared with icaritin and Epimedium extract based on an in vitro estrogen receptor mediated estrogenic assay, although it showed the strongest estrogenic activity in ovariectomized rats [10]. Accordingly, the metabolic pathway of icariin in a non-classical system, such as the digestive tract, has become more important.

Based on the pharmacokinetic study of icariin in rats, icariin is reported to be readily absorbed by the body and metabolized. The metabolism of icariin resulted in 19 phase I metabolites in rat plasma [11], but significant biliary excretion and reabsorption to the body make the pharmacokinetics of icariin more complicated [12]. Recently, Zhou et al. reported that icariin is hydrolyzed to icaritin via icariside I or II by the rat intestinal flora [13]. On the contrary, few reports on the icariin metabolism in the human body are available. Furthermore, there have been no reports regarding the human intestinal metabolism of icariin yet.

In our research group, the biotransformation of flavonoids by human intestinal bacteria has been extensively studied [14,15]. We believe the identification of intestinal metabolites of dietary flavonoids produced by human intestinal bacteria can help to explain the pharmacological effects of flavonoids that cannot be observed from the in vitro bioassay [16]. Among the beneficial biological activities of Epimedii Herba, a strong estrogenic effect of icariin drew our attention, because the molecular structure of icariin bearing two monosaccharides appeared too hydrophilic to be a strong phytoestrogen. To study the metabolism of icariin in the human intestine, the biotransformation of icariin by human intestinal microflora and the isolated bacteria *Streptococcus* sp. MRG-ICA-B, *Enterococcus* sp. MRG-ICA-E, and *Blautia* sp. MRG-PMF-1 was performed under anaerobic conditions. Based on the identified metabolites, the metabolic pathway of icariin in the human intestine was proposed for the first time in this report.

## 2. Results

### 2.1. Icariin Metabolism by Human Intestinal Microflora

**Figure 2:**
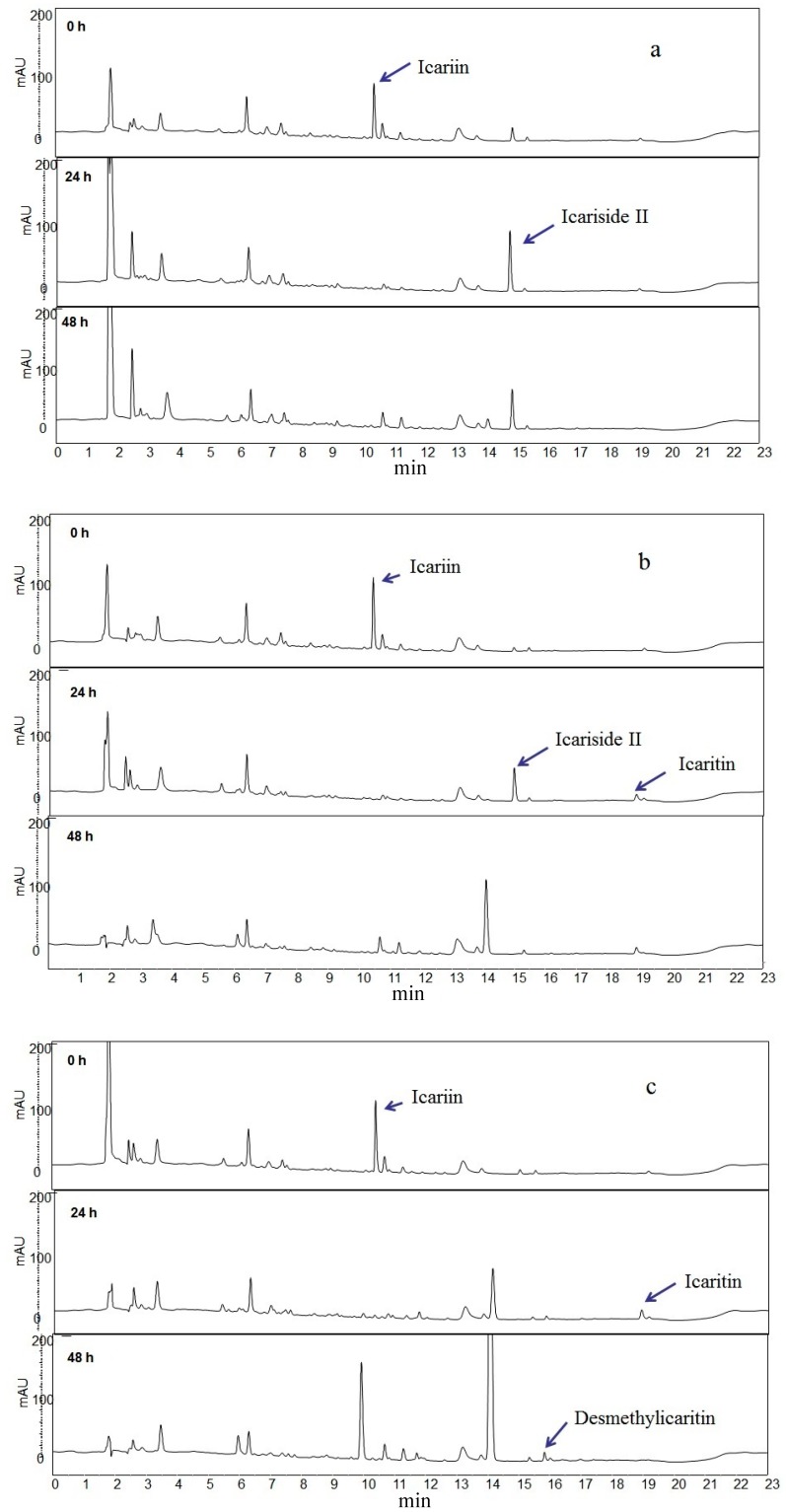
HPLC chromatograms of icariin biotransformation products. Each chromatogram was obtained from the different human intestinal microflora. Microflora **a**, **b** and **c** showed icariside II, icaritin and desmethylicaritin formation, respectively, after 48 h.

Icariin was added to the three different human intestinal microflora prepared from human fecal samples. Icariin (0.1 mM) was completely metabolized and the metabolites, such as icariside II, icaritin and desmethylicaritin, were identified from HPLC chromatograms as shown at Figure 2. Even though we cannot exclude a possible selective inhibition against specific bacteria, we could not observe any inhibitory effects of icariin, even at higher concentrations, for the growth of intestinal microflora. Therefore, we have screened three bacteria that metabolize icariin to investigate the detailed metabolic pathway.

### 2.2. Metabolites Resulting from Icariin Metabolism by Intestinal Bacteria

**Figure 3:**
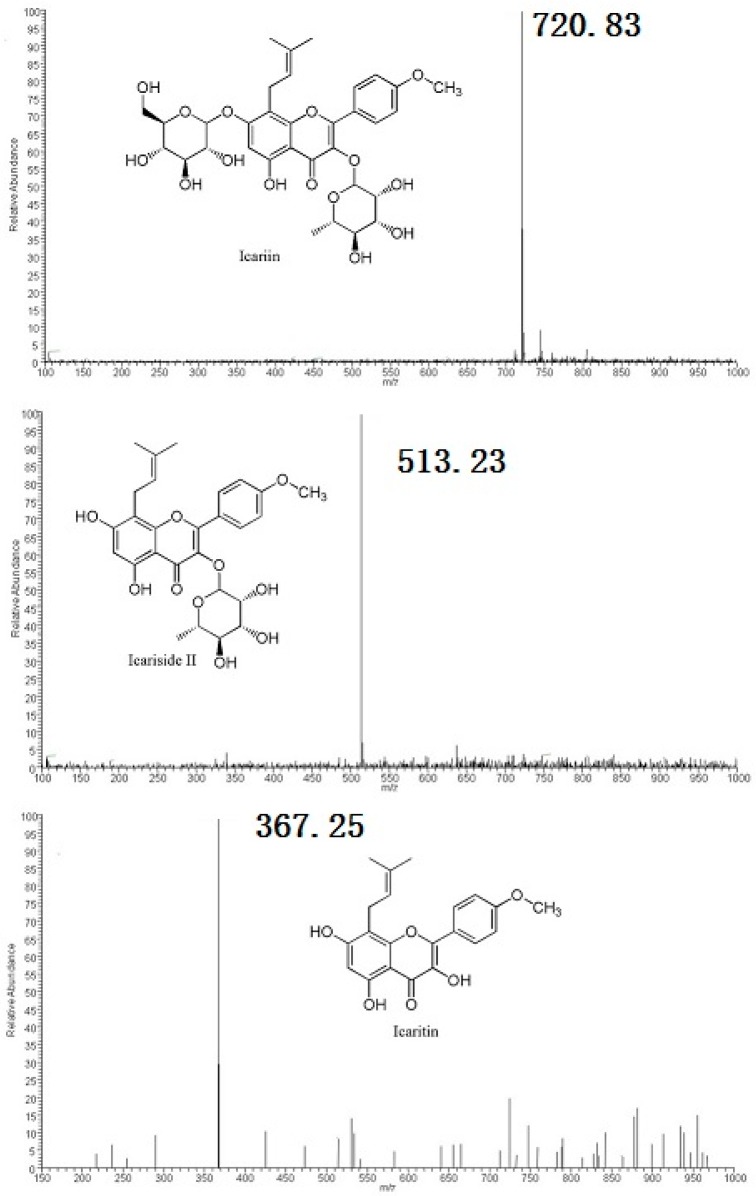

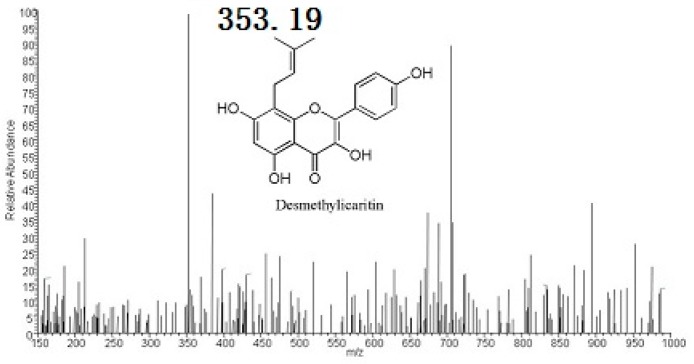
MS spectra of icariin and its metabolites. Thermo Fisher Scientific LCQ fleet instrument (Thermo Scientific, Waltham, MA, USA) was used for electrospray ionization mass spectrometry (ESI-MS) analysis. ESI condition: spray voltage, 5.4 kV; sheath gas, 15 arbitrary units; auxiliary gas, five arbitrary units; heated capillary temperature, 275 °C; capillary voltage, 27 V; and tube lens, 100 V.

**Figure 4:**
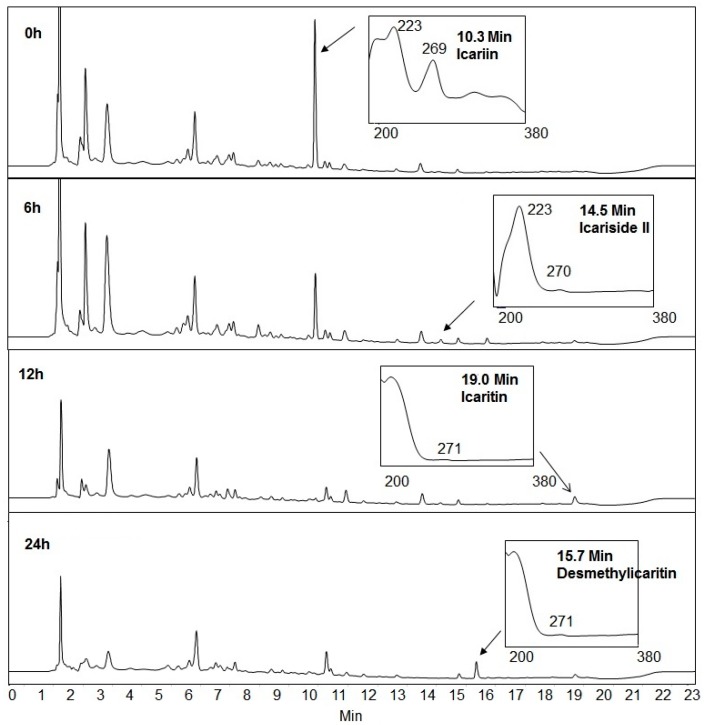
HPLC chromatogram changes at 270 nm absorption over icariin metabolism by *Blautia* sp. MRG-PMF1.

Icariin conversions by *Streptococcus* sp. MRG-ICA-B and *Enterococcus* sp. MRG-ICA-E yielded single metabolites at the retention time of 14.5 min, which were identical to HPLC and ESI-MS analyses. On the ESI-MS analysis, icariin showed a parent peak at *m*/*z* 720.89 which corresponded to the [M + CO_2_H − H]^−^ species (Figure 3). The molecular ion [M − H]^−^ peak of the metabolite at rt = 14.5 min was found at *m*/*z* 513.29 from the ESI-MS spectrum. It was smaller than that of icariin by 162 Da, which indicated the cleavage of glucose (Figure 3). Therefore, the metabolite was assigned as icariside II, which also was confirmed by comparison with the standard compound. Icariin was also metabolized by *Blautia* sp. MRG-PMF-1 (Figure 4). Along with icariside II, two new metabolites at the retention times of 19.0 min and 15.7 min were observed sequentially from the HPLC analysis. The molecular ion [M + H]^+^ peak of the metabolite at rt = 19.0 min was found at *m*/*z* 369.21 from the ESI-MS spectrum, which was smaller than that of icariside II by 144 Da, indicating the cleavage of rhamnose. The molecular ion [M − H]^−^ peak of the metabolite at rt = 15.7 min was found at *m*/*z* 353.19 from the ESI-MS spectrum, and indicated the cleavage of the methyl group from the other metabolite at rt = 19.0 min (Figure 3). Two metabolites produced only by the MRG-PMF1 strain were identified as icaritin (rt = 19.0 min) and desmethylicaritin (rt = 15.7 min), respectively.

### 2.3. Biotransformation Rate of Icariin by Intestinal Bacteria

**Figure 5:**
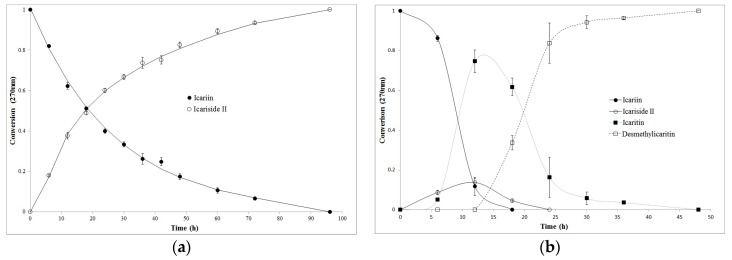
Time-dependent biotransformation of icariin by (**a**) *Streptococcus* sp. MRG-ICA-B and (**b**) *Blautia* sp. MRG-PMF-1.

Bacterial icariin conversion was studied in a time-dependent manner by HPLC analysis. Apparently, the deglycosylation of icariin by *Streptococcus* sp. MRG-ICA-B was first-order and completed in 96 h (Figure 5a). Icariin biotransformation by *Blautia* sp. MRG-PMF-1 was much faster and more complicated, and the concentrations of icariside II and icaritin were the highest at 12 h. Icariin was completely converted within a day and desmethylicaritin was the only metabolite found in the medium after two days (Figure 5b).

### 2.4. Proposed Metabolic Pathway of Icariin in Human Intestine

**Figure 6:**
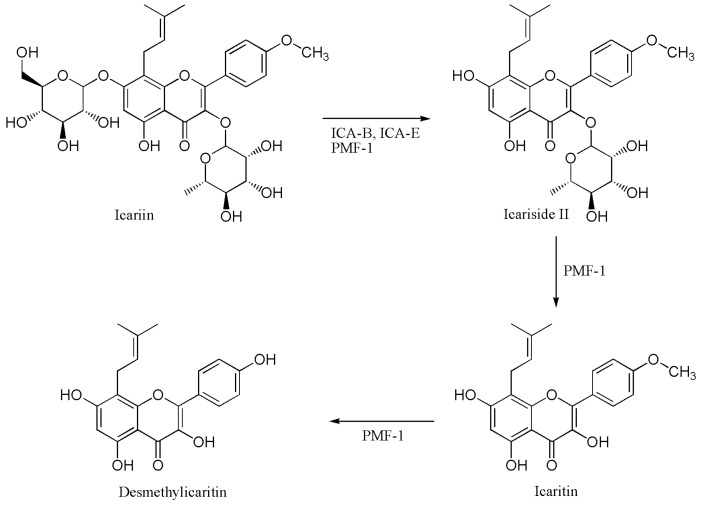
Proposed metabolic pathway of icariin in human intestine.

Icariin biotransformation by three human intestinal bacteria showed that icariin was converted to icariside II, icaritin and desmethylicaritin (Figure 6). Recently, icariin biotransformation in the rat intestine was reported, and icariside I and II were identified as metabolites resulting in icaritin [13]. However, we could not observe the formation of icariside I by human intestinal bacteria.

## 3. Discussion

Most bioactive polyphenolics are metabolized by the bacteria maintaining the symbiotic ecosystem in the human intestine, before absorption in the body [16]. We have been studying the microbial metabolism of flavonoids with isolated human intestinal bacteria to provide the biochemical information. For example, it is found that strong estrogenic *S*-equol is stereospecifically produced by human intestinal bacteria through a series of biochemical reactions including deglycosylation, reductions, and dihydroxylation from daidzin [17,18,19,20]. It is also known by many research groups, including us, that bioactive polyphenolic compounds can be biotransformed to different metabolites, depending on the microbiota harbored by the host. In the case of the daidzein metabolism, *O*-desmethylangolensin and 2*R*-(4-hydroxyphenyl)propionic acid, together with *S*-equol, were produced depending on the host microbiota [21,22]. The emerging research area of flavonoid metabolism by human intestinal bacteria provided a new paradigm in the area of pharmacokinetic studies. At the same time, it brought up the issue that many in vitro pharmacological activity measurements should be performed with the microbial metabolites which actually interact with the in vivo biochemical receptors.

In this report, the metabolism of icariin by human intestinal bacteria was investigated to provide information on whether icariin is the actual phytoestrogen responsible for biological activity in the body. Human intestinal microflora quickly metabolized 0.1 mM of icariin to icariside II within an hour as observed from the mixed cells metabolism. The concentration of icariin was chosen to represent the actual intake amount of Epimedii Herba. Similar results were observed from the icariin metabolism by rabbit intestinal flora [23]. When icariin was reacted with the isolated bacteria, icariside II was the only metabolite produced by the two bacteria *Streptococcus* sp. MRG-ICA-B and *Enterococcus* sp. MRG-ICA-E. Icariside II is also a constituent of *E. koreanum* and exhibits anti-hepatotoxic activity as strong as silybin [24]. The icariin metabolism by *Blautia* sp. MRG-PMF-1 produced further hydrolyzed products, icaritin and desmethylicaritin, which were reported to exhibit estrogenic effects by acting on the estrogen receptor [8,25]. Icaritin was recently reported to show a strong anti-cancer activity at concentrations of a few μM [26,27]. Besides, desmethylicaritin showed significant anti-adipogenesis activity [28].

Regardless of the important biological activities of icaritin and desmethylicaritin, the roles of human intestinal bacteria in the formation of these metabolites were not clear. In this report, we have confirmed the formation of icaritin and desmethylicaritin by the human intestinal bacterium *Blautia* sp. MRG-PMF1. Based on our experiments, the icariin metabolism in the human intestine looked similar to those reported from animal models. However, icariside I was not detected from our works. Furthermore, it should be also noted that individual variations in the icariin metabolism are expected due to the different host microbiota of individuals.

## 4. Materials and Methods 

The experimental protocol was evaluated and approved by the Institutional Review Board of Chung-Ang University (Approval Number: 1041078-201502-BR-029-01).

### 4.1. Chemicals and Bacteria

Icariin, icariside II and icaritin were purchased from Sigma-Aldrich (St. Louis, Mo, USA). HPLC grade acetonitrile and water were from Burdick & Jackson (Muskegon, MI, USA). Ethyl acetate and acetic acid were purchased from Fisher (Pittsburg, PA, USA). Gifu anaerobic medium (GAM) was from Nissui Pharmaceutical Co. (Tokyo, Japan). The GAM broth was prepared following the manufacturer’s instructions and every GAM plate contained 15 g/L agar in GAM broth. *N*,*N*-Dimethylformamide (99.5%, DMF) was from Samchun Pure Chemicals (Gyeonggi-do, Korea).

Human intestinal microflora and three human intestinal bacteria isolated from our laboratory, *Streptococcus* sp. MRG-ICA-B, *Enterococcus* sp. MRG-ICA-E, and *Blautia* sp. MRG-PMF1 (GenBank accession number: KT585282, KT583836, and KJ078647, respectively) were used in this experiment. Preparation of intestinal mixed cells, bacterial growth and substrate conversion experiments were performed under the anaerobic conditions, according to the published method [15,29].

### 4.2. General HPLC Methods

To monitor the reaction products, Finnigan Surveyor Plus HPLC-DAD system (Thermo Scientific) equipped with a C_18_ reversed-phase column (Hypersil GOLD 5 μm, 4.6 by 100 mm; Thermo Scientific) were employed. The mobile phase was composed of deionized water with 0.1% acetic acid (solvent A) and acetonitrile with 0.1% acetic acid (solvent B). Following the injection of 10 μL of analyte, the elution profile started with 90% of A for 1 min. The solvent gradient was then changed linearly to 40% of A over 11 min, 20% of A over next 3 min, and kept for 2 min. The eluent flow rate was 1.0 ml/min.

### 4.3. Icariin Metabolism by Human Intestinal Bacteria

Bacteria were grown to OD 0.8 at 600 nm in 99 μL of GAM broth media, and 1 μL of icariin (10 mM in DMF) was added to the each medium. After incubation for 24 h, 1 mL of ethyl acetate was added to stop the biotransformation. The mixture was then vortexed for 20 s and centrifuged (10,770× *g*) for 1 min. The supernatant (800 µL) was taken and dried under reduced pressure. The residue was then dissolved in 100 µL of DMF, and a 10 µL aliquot was injected for HPLC.

### 4.4. Structural Analyses of Icariin Metabolites by HPLC-MS

Icariin metabolites were analyzed by a HPLC-MS system. Dionex Ultimate 3000 HPLC system (Thermo Scientific) equipped with a C_18_ reversed-phase column (Kinetex, 100 × 2.1 mm, 1.7 µL, Phenomenex, Torrance, CA, USA) and a diode array detector (DAD) was coupled to a Thermo Fisher Scientific LCQ fleet instrument (Thermo Scientific) for electrospray ionization mass spectrometry (ESI-MS) analysis. The mobile phase was composed of deionized water with 0.1% formic acid (solvent A) and acetonitrile with 0.1% formic acid (solvent B). Gradient for the eluent began with 80% of A (0–1 min) and increased to 70% of A (1–5 min), to 80% of A (5–12 min), and finally to 90% of A (12–30 min). The flow rate was 0.3 mL/min. ESI condition: spray voltage, 5.4 kV; sheath gas, 15 arbitrary units; auxiliary gas, five arbitrary units; heated capillary temperature, 275 °C; capillary voltage, 27 V; and tube lens, 100 V.

### 4.5. Biotransformation Kinetics

Icariin was used as substrates for biotransformation kinetics study of *Streptococcus* sp. MRG-ICA-B and *Blautia* sp. MRG-PMF-1 in an anaerobic chamber. When the growth of bacteria in GAM broth media reached OD 1.6 (MRG-ICA-B) and 1.5 (MRG-PMF-1) at 600 nm, 100 µL of the cell culture was transferred to 10 mL of fresh GAM broth medium. Biotransformation was initiated by adding 100 µL of icariin (10 mM in DMF). To monitor the conversion rate, 100 µL of the reaction medium were taken regularly and allocated into Eppendorf tubes. The allocated media were extracted and analyzed by HPLC as described above.
